# Hypoxia Increases ATX Expression by Histone Crotonylation in a HIF-2α-Dependent Manner

**DOI:** 10.3390/ijms24087031

**Published:** 2023-04-11

**Authors:** Mengxia Qu, Yang Long, Yuqin Wang, Nan Yin, Xiaotian Zhang, Junjie Zhang

**Affiliations:** 1 The Key Laboratory of Cell Proliferation and Regulation Biology, Ministry of Education, Department of Biology, College of Life Sciences, Beijing Normal University, Beijing 100875, China; 2 Brown Foundation Institute of Molecular Medicine for the Prevention of Human Diseases, The University of Texas-Health Science Center at Houston, Houston, TX 77030, USA

**Keywords:** autotaxin, HIF-2α, hypoxia, histone crotonylation

## Abstract

Autotaxin (ATX), the key enzyme that generates lysophosphatidic acid (LPA) from lysophosphatidylcholine (LPC), is involved in tumorigenesis through the ATX-LPA axis and is regarded as a valuable target in tumor therapy. Hypoxia is a major feature of solid tumors and contributes to tumor development with striking alterations in the gene expression profile. Here, we show that hypoxia induces ATX expression in a hypoxia-inducible factor (HIF) 2α-dependent fashion in human colon cancer SW480 cells. HIF-2α is directly bound to specific hypoxia response elements (HREs) in the ATX promoter. Under hypoxic conditions, knockout or inhibition of ATX suppressed the migration of SW480 cells, which could be rescued by the addition of LPA, suggesting that the induction of ATX during hypoxia promotes cancer cell migration through the ATX-LPA axis. Further studies showed that ATX expression was induced by HIF-2α through recruiting p300/CBP, which led to crotonylation but not acetylation of histone H3 in the ATX promoter region during hypoxia. Moreover, elevation of cellular histone crotonylation levels could induce ATX expression under normoxic conditions. In conclusion, our findings reveal that ATX is induced in SW480 cells during hypoxia by histone crotonylation in a HIF-2α-dependent manner, while as a novel mechanism of ATX expression regulation, the upregulation of ATX expression by histone crotonylation is not confined to hypoxia.

## 1. Introduction

Hypoxia is a shared feature of solid tumors as tumor growth leads to insufficient local blood supply. The oxygen concentration in solid tumors is significantly lower than that in normal tissues [1], and oxygen deficiency induces hypoxia reactions mediated by hypoxia-inducible transcription factor (HIF) in tumor cells. The stability of HIF-α is regulated by oxygen concentration. Under normoxic conditions, HIF-α is modified by proline hydroxylase (PHD) at a specific proline site, and hydroxylated HIF-α is recognized by the ubiquitin E3 ligase complex and then ubiquitinated and degraded through the ubiquitin-proteasome system. Since the activity of PHD is inhibited at low oxygen concentrations, during hypoxia HIF-α is stable and not modified by hydroxylation [2], then intracellular HIF-α and HIF-β form a heterodimer that binds to the hypoxia-response element (HRE) located upstream of a series of genes to regulate the expression of various genes related to cell proliferation, angiogenesis, metabolic modulation, and many other processes [3,4].

Previous studies have shown that hypoxia can lead to changes in chromatin modification in cells [5]. P300 and CREB-binding protein (CBP) are two paralogs with strong histone acetylsinotransferase (HAT) activity. Under hypoxic conditions, p300 and CBP can interact with HIF-1α or HIF-2α as coactivators to regulate gene expression by their histone acetylation activity over a few HIF target promoters [6]. P300 and CBP also possess histone crotonyltransferase (HCT) activity. Histone lysine crotonylation (Kcr) has been implicated in various physiological and pathological processes, such as differentiation, tissue injury, and tumorigenesis [7]. The p300/CBP-catalyzed Kcr can directly stimulate transcription, but its role in the hypoxic response remains unknown.

Autotaxin (ATX) is a secreted glycoprotein with lysoPLD activity. Its main biochemical function is to convert lysophosphatidylcholine (LPC) to lysophosphatidic acid (LPA) [8]. The biological function of ATX is mainly achieved by catalyzing the production of the bioactive lipid LPA, which binds to its specific receptors on the cell membrane to regulate many aspects of cell function, including proliferation and migration. Studies in recent years have shown that the ATX-LPA axis has a wide range of biological functions and is not only involved in angiogenesis and neural development but also plays important roles in obesity-related diseases, immune regulation, and tumorigenesis [9,10,11]. ATX was first isolated from the conditioned medium of human melanoma cells as a motility-stimulating factor [12]. Exogenous expression of ATX in NIH3T3 cells significantly promotes tumorigenic and migratory abilities [13]. ATX is highly expressed in lung cancer cells DMS273, ovarian cancer cells SKOV3, and colon cancer cells Colo320, as well as in most glioma cells, and the expression of ATX greatly increases the motility of tumor cells [14]. ATX is expressed in tumors such as non-small cell lung cancer, breast cancer, prostate cancer, liver cancer, thyroid cancer, and glioma, and is one of the 40 major upregulated genes associated with tumor migration [15,16]. Since the expression of ATX in tumor cells plays an important role in the occurrence and development of tumors, especially in tumor migration, ATX is considered an important target for tumor therapy. In addition, ATX is a biomarker for tumor diagnosis or prognosis. High activity of ATX in the ascites of ovarian cancer patients accelerates tumor development [17]. The levels of ATX decrease in the serum of prostate cancer patients after surgery and can be used as a prognostic indicator for prostate cancer [18]. It has been suggested that ATX can be used as a prognostic indicator for judging the prognosis of patients with melanoma [19]. In addition, the serum level of ATX is significantly upregulated in patients with liver fibrosis, and ATX is thought to play a key role in the development of liver cancer [20]. Therefore, ATX is not only an important target for tumor therapy but also an important marker of tumor development.

It is generally accepted that ATX is expressed in various cancer cells, but ATX expression levels are very low or undetectable in some epithelial cancer cells, such as human colon cancer cells SW480 and DLD1 and cervical cancer cells Hela [14]. Considering that ATX is closely related to the development of several solid tumors characterized by hypoxia, this study investigates the regulation of ATX expression under hypoxia. It was found that ATX was induced in SW480, DLD1, and Hela cells under hypoxic conditions. We demonstrate that the hypoxic induction of ATX in SW480 cells was in a HIF-2α-dependent manner, and that p300/CBP was recruited to the ATX promoter, contributing to the hypoxic ATX induction through the p300/CBP-mediated histone crotonylation. Further studies suggest that histone crotonylation is a novel mechanism of ATX expression regulation not only in hypoxic but also in normoxic conditions.

## 2. Results

### 2.1. Hypoxia Increases ATX Expression in Cancer Cells

**Figure 1:**
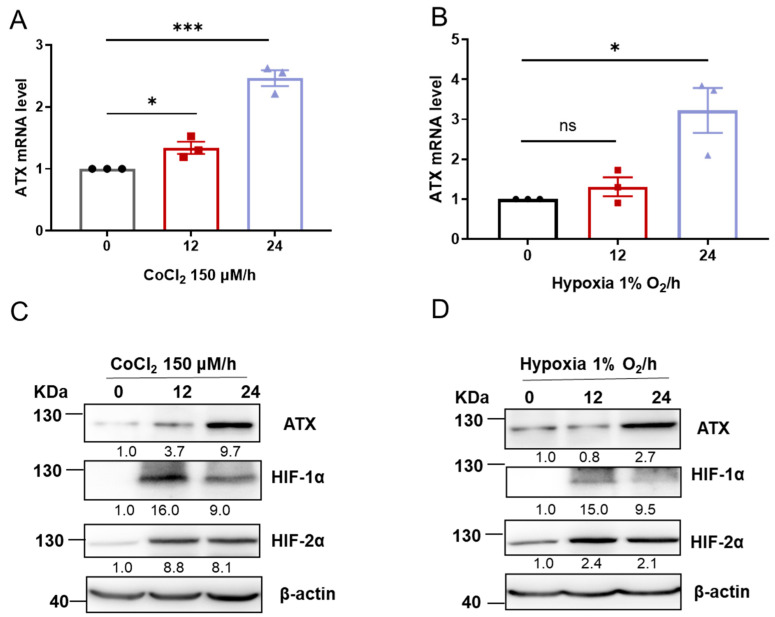
ATX induction by CoCl_2_ treatment and hypoxia. (**A**,**B**) RT-qPCR analysis of ATX mRNA levels in SW480 cells treated with CoCl_2_ (150 μM) (**A**) or exposed to hypoxia (1% O_2_) for 0, 12, or 24 h (**B**). (**C**,**D**) Immunoblot assays of ATX, HIF-1α and HIF-2α expression levels in SW480 cells treated with CoCl_2_ (150 μM) (**C**) or exposed to hypoxia (1% O_2_) (**D**) for 0, 12, or 24 h. The protein levels were normalized and quantified by Image J 1.8.0. The data shown are the mean ± SEM of *n* = 3 independent experiments. *p*-values were calculated using two-sided unpaired Student’s *t* tests (**A**,**B**). ns, not significant; * *p* < 0.05; *** *p* < 0.001.

Computer analysis (http://jaspar.genereg.net/) showed that there are HREs in the human ATX promoter region, suggesting that ATX may be a target gene of HIF (see below). To test our conjecture, human colon cancer SW480 cells were treated with (150 μM) to mimic hypoxic conditions. Quantitative reverse transcription–polymerase chain reaction (RT-qPCR) and western blot assays showed that ATX expression was upregulated by CoCl_2_ treatment in a time-dependent manner (Figure 1A,C). When SW480 cells were cultured under hypoxic conditions (1% O_2_), ATX expression was also increased in a time-dependent manner (Figure 1B,D). The elevations in HIF-1α and HIF-2α protein levels indicated that hypoxia reactions were successfully induced by CoCl_2_ and 1% O_2_ treatment (Figure 1C,D). ATX induction was also observed in human colon cancer DLD1 and Hela cells treated with CoCl_2_ or cultured under hypoxic conditions (1% O_2_) (Figure S1). These data indicate that hypoxia can increase ATX expression in certain cancer cells.

### 2.2. Induction of ATX Promotes Cancer Cell Migration during Hypoxia

**Figure 2:**
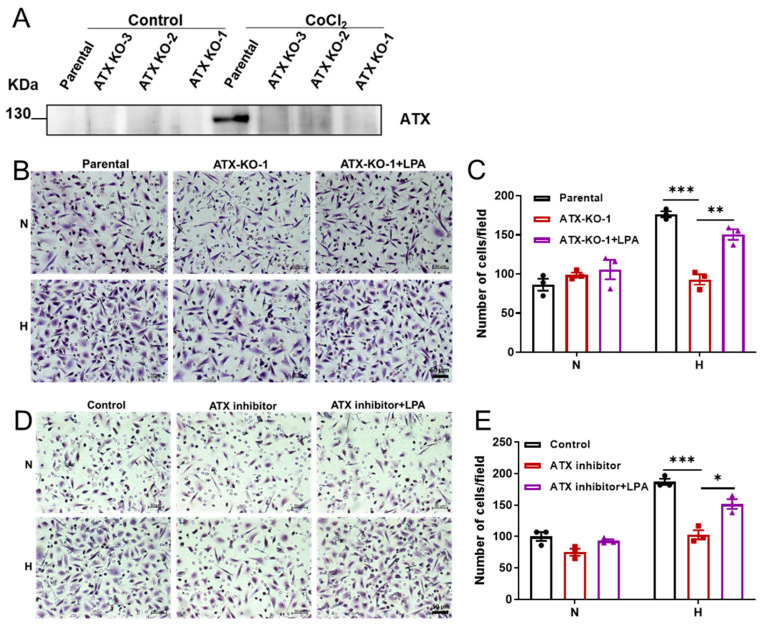
The impact of ATX knockout or inhibition on cell migration during hypoxia. (**A**) Immunoblot assays of ATX levels expressed from SW480 parental cells and ATX–KO cells treated with or without CoCl_2_ (150 μM) for 24 h. (**B**,**C**) Transwell assays were performed with SW80 parental cells, ATX-KO-1 cells, and ATX-KO-1 cells in the presence of LPA (2 µM) as indicated under normoxic (21% O_2_, N) or hypoxic (1% O_2_, H) conditions for 24 h (**B**), and then the relative migration was calculated (**C**). (**D**,**E**) Transwell assays were performed with SW80 cells alone or with SW80 cells in the presence of the ATX inhibitor PF8380 (10 µM) with/without LPA (2 µM) as indicated under normoxic (21% O_2_, N) or hypoxic (1% O_2_, H) conditions for 24 h (**D**), and then the relative migration was calculated (**E**). Images of cells on the lower surface of the upper chamber (scale bar = 50 μm) were taken 24 h after the cells were seeded into the upper chamber. Scale bar, 50 μm (**B**,**D**). The relative migration was calculated by counting the migrated cells, and data were obtained from three randomly chosen fields. (**C**,**E**). The data shown are the mean ± SEM of *n* = 3 independent experiments. *p*-values were calculated using two-sided unpaired Student’s *t* tests (**C**,**E**). * *p* < 0.05; ** *p* < 0.01, *** *p* < 0.001.

ATX performs its biological function mainly by catalyzing the production of LPA and then activating LPA receptors and their downstream pathways. The ATX-LPA axis has a wide range of cellular functions, especially to promote cell migration. Transwell assays were performed to determine the impact of ATX induction on cellular migration during hypoxia. First, ATX-knockout cell lines (ATX-KO-1/2/3) were constructed using the CRISPR-Cas9 method with SW480 as parental cells (Figure 2A and Figure S2). Second, Transwell chamber assays were carried out with ATX-KO-1 cells and SW480 parental cells, demonstrating that hypoxia increased the migration of parental SW480 cells across the Transwell membrane by 1.5-fold. Compared with parental SW480 cells, the migration ability of ATX-KO-1 cells decreased significantly under hypoxic conditions, and the administration of LPA in the culture medium restored the migratory ability of ATX knockout cells during hypoxia (Figure 2B,C). Finally, we determined the impacts of ATX inhibitors on cellular migration during hypoxia. The migratory ability of SW480 cells under hypoxic conditions was suppressed by PF8380, a specific inhibitor of ATX, which was rescued by the addition of LPA (Figure 2D,E). These data suggest that the induction of ATX expression promotes SW480 cell migration under hypoxic conditions.

### 2.3. Hypoxic Induction of ATX Is HIF-2α Dependent

**Figure 3:**
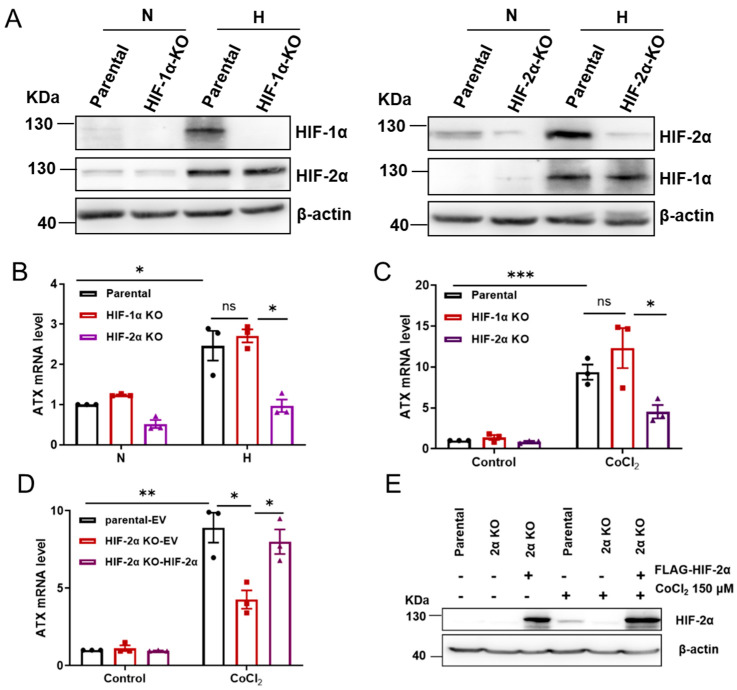
The hypoxic induction of ATX is HIF-2α dependent. (**A**) Immunoblot assays of HIF-1α and HIF-2α expression levels in parental, HIF-1α–KO, and HIF-2α–KO SW480 cells exposed to normoxia (N) or hypoxia (1% O_2_, H) for 24 h. (**B**) RT-qPCR analysis of ATX mRNA levels in parental, HIF-1α–KO, and HIF-2α–KO SW480 cells exposed to normoxia (N) or hypoxia (1% O_2_, H) for 24 h. (**C**) RT-qPCR analysis of ATX mRNA levels in parental, HIF-1α–KO, and HIF-2α–KO SW480 cells treated with or without CoCl_2_ (150 μM) for 24 h. (**D**,**E**) SW480 cells transfected with FLAG-vector or FLAG-HIF-2α vector and treated with CoCl_2_ (150 μM) for 24 h. ATX mRNA levels were detected by RT-qPCR (**D**), and HIF-2α protein levels were detected by immunoblotting (**E**). The data shown are the mean ± SEM of *n* = 3 independent experiments. *p*-values were calculated using two-way ANOVA with Tukey’s *t* test (**B**–**D**). ns, not significant; * *p* < 0.05; ** *p* < 0.01; *** *p* < 0.001.

HIFs are the key transcription factors that regulate cellular responses to hypoxia. It has been reported that HIF-1α and HIF-2α have a similar domain architecture and are regulated in a similar manner, but there are distinct differences in their target genes [21]. To determine whether the hypoxia-induced expression of ATX is HIF-1α- or HIF-2α dependent, HIF-1α-knockout (HIF-1α-KO) and HIF-2α-knockout (HIF-2α-KO) cell lines were constructed with the CRISPR-Cas9 method with SW480 cells as parental cells (Figure 3A and Figure S3). The induction of ATX expression either by hypoxia (1% O_2_) or by CoCl_2_ treatment was abolished in HIF-2α-KO cells but was not affected in HIF1α-KO cells (Figure 3B,C). In HIF2α-KO cells, ATX induction by CoCl_2_ treatment could be restored by the complementary expression of HIF-2α (Figure 3D,E). When HIF-1α and HIF-2α were knocked down respectively with their specific siRNAs in Hela cells, it was found that the ATX induction by CoCl_2_ treatment was suppressed by the knockdown of HIF-2α but not HIF-1α (Figure S4). Taken together, these results suggest that the hypoxic induction of ATX is dependent on HIF-2α but not HIF-1α.

### 2.4. HIF-2α Binds to the HREs in the ATX Promoter

**Figure 4:**
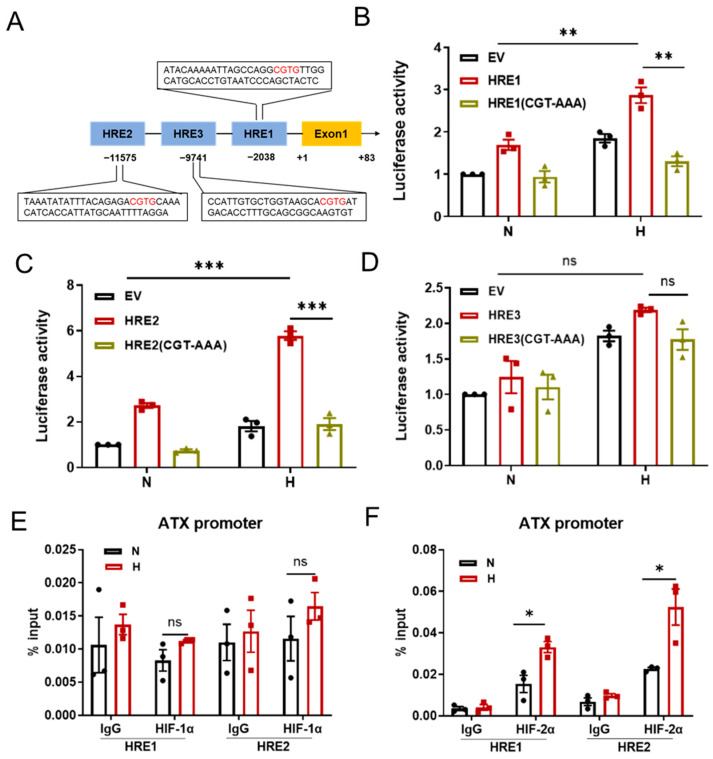
HIF-2α directly binds to the HREs in the ATX promoter. (**A**) A simplified schematic showing the putative HREs with conserved core sequences in red in the human ATX promoter region; +1 indicates the translational start site. (**B**–**D**) SW480 cells were transfected with the luciferase reporter plasmid with the indicated HRE or HRE mutant for 24 h, and then the cells were exposed to normoxia (N) or hypoxia (1% O_2_, H) for 24 h. Each cell lysate was collected, and the luciferase activity was detected. Firefly luciferase activity (Fluc) was measured and normalized to Renilla luciferase activity (Rluc). (**E**,**F**) SW480 cells were cultured under normoxic (N) or hypoxic conditions (1% O_2_, H) for 24 h and then subjected to ChIP assays with anti-HIF-1α antibody (**E**) or anti-HIF-2α antibodies (**F**) to detect the binding abilities of HIF-1α and HIF-2α to HRE1 or HRE2, respectively. IgG was used as a control. The data shown are the mean ± SEM of *n* = 3 independent experiments. *P*-values were calculated using a two-way ANOVA with Tukey’s *t* test (**B**–**D**) and two-sided unpaired Student’s *t* tests (**E**,**F**). ns, not significant; * *p* < 0.05, ** *p* < 0.01, *** *p* < 0.001.

HRE refers to the cis response element recognized by HIFs located in the promoter region of the HIF target gene. Computer analysis indicated that there are three putative sites containing HREs in the human ATX promoter (Figure 4A). We further identified the functional HREs in the ATX promoter with a dual-luciferase reporter assay. An approximately 60 bp oligonucleotide sequence containing one copy of each ATX HRE was inserted upstream of the firefly luciferase (FLuc) coding sequence to construct the corresponding ATX HRE reporter plasmid. SW480 cells were cotransfected with the ATX HRE reporter plasmid together with a control reporter, pSV40-Renilla, in which Renilla luciferase is constitutively expressed, and then exposed to normoxic or hypoxic conditions (1% O_2_) for 24 h. Both ATX HRE1 and HRE2 were able to significantly increase FLuc activity in hypoxic SW480 cells. The hypoxia-induced FLuc activity was completely abolished when the core sequence 5′-CGT-3′ within either HRE1 or HRE2 was mutated to 5′-AAA-3′ (Figure 4B,C). Meanwhile, ATX HRE3 was not functional in the hypoxic reaction (Figure 4D). A chromatin immunoprecipitation (ChIP) assay was performed to detect the binding of HIF-1/2α to ATX HREs in SW480 cells under hypoxic conditions (1% O_2_). It was found that hypoxia treatment promoted the binding of HIF-2α, but not HIF-1α, to the HRE1 and HRE2 sites in the ATX promoter (Figure 4E,F). These data indicate that during hypoxia, HIF-2α directly binds to HRE1 and HRE2 in the ATX promoter to induce their transcription.

### 2.5. p300/CBP Plays a Role in the Hypoxic Induction of ATX

**Figure 5:**
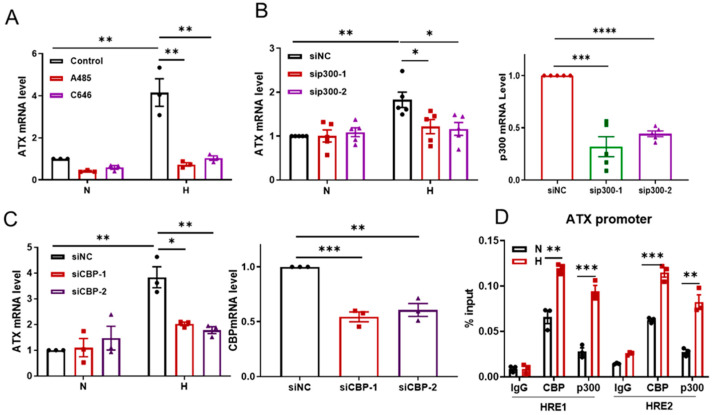
p300/CBP participates in ATX induction during hypoxia. (**A**) SW480 cells were cultured under normoxic (N) or hypoxic (1% O_2_, H) conditions with A485 (200 nM) or C646 (25 μM) treatment for 24 h. ATX mRNA levels were detected using RT-qPCR. (**B**,**C**) SW480 cells were transfected with p300 siRNA or CBP siRNA and then cultured under normoxic (N) or hypoxic (1% O_2_, H) conditions for 24 h. ATX, CBP, or p300 mRNA levels were detected with RT-qPCR. (**D**) SW480 cells were cultured under hypoxic conditions for 24 h and then subjected to ChIP assays with an anti-CBP antibody or an anti-p300 antibody to detect the binding abilities of CBP and p300 to HRE1 or HRE2, respectively. IgG was used as a control. And the data shown are the mean ± SEM of *n* = 3/5 independent experiments. *p*-values were calculated with a two-way ANOVA with Tukey’s *t* test (**A**–**C**) and two-sided unpaired Student’s *t* tests (**D**). * *p* < 0.05; ** *p* < 0.01; *** *p* < 0.001; **** *p* < 0.0001.

Histone acetyltransferase p300/CBP has been reported as a coactivator to potentiate hypoxic induction of HIF-α target genes [21]. To test whether p300/CBP is involved in the hypoxic induction of ATX, SW480 cells were cultured under hypoxic conditions (1% O_2_) with or without the presence of C646 and A485, two small molecule inhibitors of p300 or p300/CBP. Inhibition of p300 or p300/CBP activity led to significant suppression of ATX induction by hypoxia (Figure 5A). Similar impact of the p300 inhibitor on hypoxic ATX induction was observed in Hela cells (Figure S5). Next, p300 and CBP were knocked down using their specific siRNAs in SW480 cells, and then the cells were subjected to hypoxic treatment (1% O_2_). Either p300 or CBP knockdown inhibited the hypoxic induction of ATX (Figure 5B,C). Moreover, ChIP assays were performed with p300- or CBP-specific antibodies in SW480 cells under hypoxic conditions (1% O_2_). Under hypoxic conditions, p300/CBP is directly bound to the HRE1 and HER2 sites in the ATX promoter (Figure 5D). These data suggest that during hypoxia, p300/CBP is recruited to the ATX promoter by HIF-2α to activate ATX transcription.

### 2.6. Histone Crotonylation Contributes to the Hypoxic Induction of ATX

**Figure 6:**
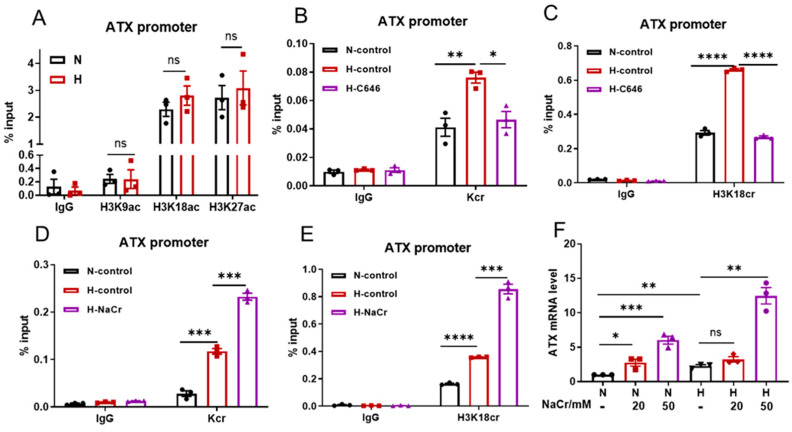
Histone crotonylation contributes to the hypoxic induction of ATX. (**A**) SW480 cells were cultured under normoxic (N) or hypoxic (1% O_2_, H) conditions for 24 h, and then subjected to ChIP assays with anti-H3K9ac, anti-H3K18ac, or anti-H3K27ac antibodies to detect the enrichment of the indicated histone modifications in the ATX promoter. (**B**–**E**) SW480 cells were cultured under normoxic (N) or hypoxic (1% O_2_, H) conditions in the absence or presence of the p300 inhibitor C646 (25 μM) (**B**,**C**) or NaCr (50 mM) (**D**,**E**) for 24 h and then subjected to ChIP assays with anti-Kcr (**B**,**D**) or anti-H3K18cr (**C**,**E**) antibodies to detect the enrichment of the indicated histone modification in the ATX promoter. The PCR amplification of immunoprecipitated DNA fragments in ChIP assays (**A**–**E**) was performed with primers for site 1. (**F**) SW480 cells were cultured under normoxic (N) or hypoxic (1% O_2_, H) conditions in the presence of NaCr (0, 20, or 50 mM) for 24 h. ATX mRNA levels were measured using RT-qPCR. And the data shown are the mean ± SEM of *n* = 3 independent experiments. *p*-values were calculated using two-sided unpaired Student’s *t* tests (**A**–**E**) and a two-way ANOVA with Tukey’s *t* test (**F**). ns, not significant; * *p* < 0.05; ** *p* < 0.01; *** *p* < 0.001; **** *p* < 0.0001.

P300/CBP have HAT and HCT activities to catalyze histone lysine acetylation (Kac) and histone lysine crotonylation (Kcr), respectively [22]. The roles of Kac and Kcr in hypoxic induction of ATX were further explored. Since histone acetylation usually occurs at sites such as H3K9, H3K18, and H3K27 under hypoxic conditions [6], ChIP assays were performed to probe whether H3K9ac, H3K18ac, and H3K27ac were involved in the hypoxic induction of ATX. Our ChIP results showed that there were no differences in the acetylation levels at these histone sites in the ATX promoter between the normoxic and hypoxic SW480 cells (Figure 6A and Figure S6). Regarding histone crotonylation, ChIP assay results with an anti-Kcr antibody indicated that in hypoxic SW480 cells (1% O_2_), compared to cells under normoxic (21% O_2_) conditions, there were significantly higher general Kcr levels in the ATX promoter, which could be decreased by treatment with the p300 inhibitor C646 (Figure 6B). With an antibody specific for H3K18cr, ChIP assays revealed that the H3K18 crotonylation (H3K18cr) levels in the ATX promoter were increased in hypoxic SW480 cells, which were also suppressed by C646 treatment (Figure 6C). Moreover, in the presence of crotonate (NaCr), a crotonyl-CoA precursor, the general Kcr and H3K18cr levels in the ATX promoter were further increased (Figure 6D,E), and the induction of ATX expression was further promoted in hypoxic SW480 cells (Figure 6F). All of these results suggest that p300/CBP-mediated histone crotonylation contributes to the hypoxic induction of ATX. Notably, ATX expression was also increased by crotonate (NaCr) under normoxic conditions (Figure 6F), which means that the promotion of ATX expression by histone crotonylation is not a mechanism confied to the hypoxia reaction.

### 2.7. Histone Crotonylation Increases ATX Expression under Normoxic Conditions

**Figure 7:**
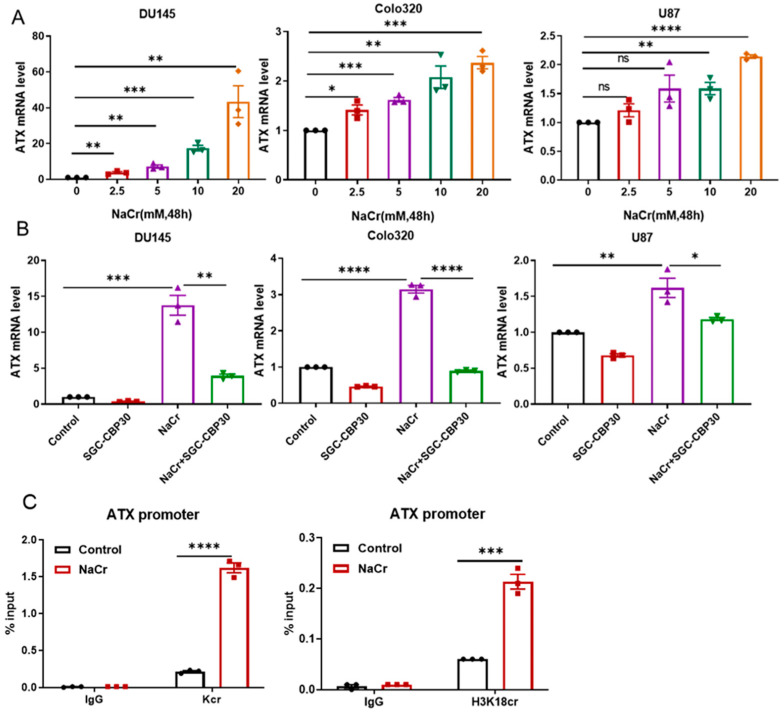
Histone crotonylation promotes ATX expression under normoxic conditions. (**A**) DU145, Colo320, and U87 cells were cultured under normoxic (N) conditions in the absence or presence of NaCr (0, 2.5, 5, 10, or 20 mM) for 48 h. (**B**) DU145, Colo320, and U87 cells were cultured under normoxic (N) conditions in the absence or presence of NaCr (10 mM) and/or the p300/CBP inhibitor SGC-CBP30 (25 µM) as indicated for 48 h. ATX mRNA levels were measured using RT-qPCR (**A**,**B**). (**C**) DU145 cells were cultured under normoxic conditions in the absence or presence of NaCr (10 mM) for 48 h and then subjected to ChIP assays with anti-Kcr and anti-H3K18cr antibodies. The PCR amplifications of immunoprecipitated DNA fragments in ChIP assays were performed with primers for site 1. The data shown are the mean ± SEM of *n* = 3 independent experiments. *p*-values were calculated using two-sided unpaired Student’s *t* tests (**A**–**C**). * *p* < 0.05; ** *p* < 0.01; *** *p* < 0.001; **** *p* < 0.0001.

To further probe the universal role of histone crotonylation in ATX induction, a panel of human cancer cells with relatively high-level endogenous ATX expression, including DU145, Colo320, and U87 cells, were cultured in the presence or absence of crotonate (NaCr) under normoxic conditions. ATX expression was upregulated in these cells by NaCr in a dose-dependent manner (Figure 7A). The induction of ATX by NaCr was inhibited by the addition of SGC-CBP30, an inhibitor of p300/CBP (Figure 7B). ChIP assay results with either anti-Kcr or anti-H3K18cr antibodies demonstrated that the histone crotonylation levels were increased in the ATX promoter by NaCr (10 mM) treatment (Figure 7C). Therefore, the induction of ATX by histone crotonylation occurs under both hypoxic and normoxic conditions. However, when colon cancer Colo320 cells and melanoma A375 cells with high levels of endogenous ATX expression were subjected to CoCl_2_ or hypoxia treatment, the ATX expression levels in these cells were significantly decreased, indicating that there is a noteworthy difference in the regulation of ATX expression by hypoxia among different cells (Figure S7).

## 3. Discussion

Hypoxia is a common feature of solid tumors, which are less well oxygenated than normal tissues. The cellular response to hypoxia is mainly mediated by hypoxia-inducible factors (HIFs), a family of heterodimers consisting of an oxygen-sensitive HIFα subunit and an oxygen-insensitive HIF-β subunit. Under hypoxic conditions, the HIFα/β heterodimer regulates the expression of multiple genes involved in cell proliferation, angiogenesis, and metabolic regulation, which facilitates the adaptation and progression of cancer cells [3,4]. HIF-1α and HIF-2α have similar characteristics, such as sensitivity to oxygen, interaction with HIFβ, and binding to HRE motifs of hypoxia-responsive genes to regulate transcriptional activation. However, the expression levels of HIF-1α and HIF-2α are different in various tissues [23,24,25,26,27]. HIF-2α is most abundantly expressed in adult vascular endothelial cells, lungs, placenta, and heart, and HIF-1α is ubiquitously expressed in all analyzed mammalian tissues and cell types [24,27,28,29]. Moreover, HIF-1α and HIF-2α have different specificities for transcriptional targets. For example, HIF-1α is effective in stimulating the expression of glycolytic enzymes such as lactate dehydrogenase A (LDH-A) and carbonic anhydrase IX (CAIX); in contrast, HIF-2α is more potent for the EPO gene and genes involved in iron metabolism, while another group of genes, including VEGF and GLUT-1, are regulated by both HIF-1α and HIF-2α [30].

ATX is the key enzyme to produce LPA, a bioactive lipid that acts through its receptors on the surface of the cell membrane. Studies in recent years have shown that the ATX-LPA axis plays a role in tumorigenesis, especially in tumor metastasis [31]. In this study, we demonstrate that ATX could be induced by hypoxia, a common feature of solid tumors. Two functional HRE motifs were identified in the ATX promoter, and the hypoxic induction of ATX is dependent on HIF-2α but not HIF-1α, indicating that ATX is a target gene of HIF-2α. In a previous study, it was reported that hepatitis C virus infection increased ATX expression in hepatocellular carcinoma cells via HIF-1α [32]. The reasons for this discrepancy may lie in the differences in cell types and stress conditions.

In this study, it was found that the induction of ATX promoted cancer cell migration under hypoxic conditions. In accordance with our results, it has been reported that hypoxia increases the expression of ATX while decreasing the expression of the LPA-degrading enzymes LPP1 and LPP3 in certain cancer cells to promote cell invasion [33]. As a multifunctional signaling pathway, the ATX-LPA axis also has antiapoptotic and proangiogenic functions. Hypoxia-induced ATX expression may promote hypoxia resistance in tumor cells and contribute to the formation and development of solid tumors.

HIFs recruit coactivators to modify chromatin structures, thereby facilitating gene expression regulation by transcriptional mechanisms. P300 and CBP are well-known HIF coactivators [6], possessing histone acetyltransferase (HAT) and histone crotonyltransferase (HCT) activities to catalyze histone lysine acetylation (Kac) and histone lysine crotonylation (Kcr). It has been reported that p300/CBP-catalyzed histone Kcr can directly stimulate transcription. Histone Kcr is associated with physiological and pathological processes such as differentiation, tissue damage, viral infections, tumorigenesis, and neurodegenerative diseases [22]. However, the role of histone Kcr in the cellular hypoxia response remains unclear. Our findings indicate that p300/CBP can be recruited to the ATX promoter by HIF-2α and that p300/CBP-catalyzed histone Kcr, but not Kac, mainly contributes to the hypoxic induction of ATX in SW480 cells. In the presence of crotonate (NaCr), a crotonyl-CoA precursor, the histone Kcr levels in the ATX promoter were further increased, and the upregulation of ATX expression was much more enhanced under hypoxic conditions. Recent studies have revealed that histone lysine crotonylation is involved in the development of some solid tumors, including liver, stomach, kidney, thyroid, esophagus, colon, pancreas, and lung carcinomas [7]. The correlation between ATX expression and Kcr levels in clinical solid tumor samples should be detected in future studies.

Moreover, in some cancer cells with relatively high endogenous ATX levels, it is interesting to note that ATX expression can be further upregulated by histone crotonylation in the presence of NaCr under normoxic conditions, suggesting that the induction of ATX expression by histone crotonylation is not confined to hypoxia. ATX upregulation by histone crotonylation under normoxic conditions is also dependent on p300/CBP. However, in Colo320 cells and melanoma A375 cells that express robust ATX, ATX expression levels were significantly decreased by hypoxia (Figure S7), indicating that there is a remarkable difference in the regulation of ATX expression by hypoxia among different cells. In other words, the hypoxic induction of ATX may be a feature of epithelial cancer cells with low or undetectable endogenous ATX levels. 

**Figure 8:**
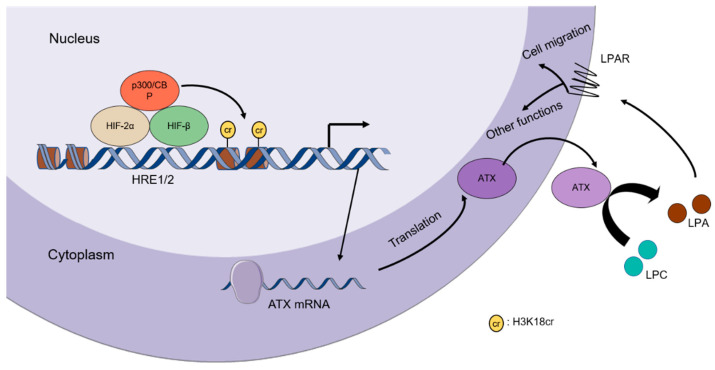
Schematic representation of the proposed ATX expression regulation mechanisms during hypoxia. Under hypoxic conditions, HIF-2α is stabilized and interacts with HIF-β to form a heterodimer, which can bind to the functional hypoxia-response elements (HREs) in the ATX promoter and recruit p300/CBP to the ATX promoter. p300/CBP-mediated histone crotonylation induces ATX expression, leading to the elevation of extracellular LPA levels to promote cell migration through LPA receptors on the cell surface. LPAR, LPA receptor.

In summary, our data demonstrate that hypoxia increases ATX expression by histone crotonylation in a HIF-2α-dependent manner in certain epithelial cancer cells (Figure 8). In addition to solid tumors, hypoxia also occurs in other human pathological conditions such as stroke, myocardial infarction, and chronic kidney disease [6]. The regulation of ATX and its function under these pathological conditions remain unknown. 

## 4. Materials and Methods

### 4.1. Chemicals and Reagents

The p300 inhibitor C646 (328968-36-1), P300/CBP inhibitor A485 (1889279-16-6), SGC-CBP30 (1613695-14-9), and crotonate (NaCr) (107-93-7) were purchased from MedChemExpress (Monmouth Junction, NJ, USA). CoCl_2_ (7646-79-9) was obtained from Sigma-Aldrich (Buchs, Switzerland). Antibodies against HIF-1α (NB100-105) and HIF-2α (NB100-122) were purchased from Novus Biologicals (Littleton, CO, USA). Mouse immunoglobin IgG protein (sc-2025) and an antibody against β-actin (sc-8432) were purchased from Santa Cruz (Santa Cruz, CA, USA). Antibodies against p300 (ab14984), H3K9ac (ab4441), H3K18ac (ab1191), and H3K27ac (ab4729) were obtained from Abcam (Cambridge, UK). Antibodies against CBP (7389) were purchased from Cell Signaling Technology (Danvers, MA, USA). Antibodies against Kac (PTM-105), Kcr (PTM-501), and H3K18cr (PTM-540) were obtained from PTM Biolab (Hang Zhou, China). Antibodies against histone H3 (30005ES50) were purchased from Yeasen (Shang Hai, China). The ATX primary antibody was generated as described previously [34]. ChIP-Grade Protein A/G Magnetic Beads (26162), Lipofectamine 3000 (L3000015), and Lipofectamine RNAi MAX transfection reagent (13778150) were purchased from Thermo Fisher Scientific (Bremen, Germany).

### 4.2. Cell Culture and Cell Treatment

SW480 cells (from the National Infrastructure of Cell Line Resource, China) were cultured in IMDM medium (Macgene, Beijing, China, #CM10016), DLD1 and Colo320 cells (a gift from Dr. Yu Shang, Beijing Normal University) were cultured in RPMI1640 medium (Macgene, Beijing, China, #CM17106), DU145, U87, Hela, and A375 cells (from the National Infrastructure of Cell Line Resource, China) were cultured in DMEM medium (Macgene, Beijing, China, #CM10017), supplemented with 10% FBS (TIANHANG, Hang Zhou, China, #11012-8611), 100 U/mL penicillin, and 0.1 mg/mL streptomycin (Gibco, Grand Island, NY, USA, #15140122) at 37 °C in a humidified 5% CO_2_ atmosphere cell culture incubator. Hypoxic cells were placed in a tri-gas incubator (Thermo Fisher Forma 3131, Bremen, Germany) and flushed with a gas mixture of 1% O_2_, 5% CO_2,_ and balanced N_2_. To mimic hypoxic conditions, cells were treated with CoCl_2_ (150 μM). The p300 HAT inhibitor C646 and the p300/CBP HAT inhibitor A485 were used at final concentrations of 25 μM and 0.2 μM, respectively, either alone or in combination with hypoxia (1% O_2_) for 24 h. Cells were treated with 0, 2.5, 5, 10, 20, or 50 mM crotonate (NaCr) for 36 h or 48 h to induce crotonylation.

### 4.3. CRISPR/Cas9 Assay

sgRNAs targeting HIF-1α, HIF-2α, and ATX were designed. The sgRNA target sequences were as follows: 5′-CCATCAGCTATTTGCGTGTG-3′ (HIF-1α-KO); 5′ CTTCCGGCATCAAAGAAGA -3′ (HIF-2α-KO); 5′-CTTTCCAAGAATCCTCGACA-3′ (ATX-KO-1); 5𠄲- GTGGCACACACTCTCCCTACATG -3′ (ATX-KO-2/3). DNA oligos for sgRNA were synthesized and cloned into pSpCas9(BB)-2A-GFP (termed PX458) (Addgene, Watertown, MA, USA, Plasmid #48138) to create the CRISPR-Cas9-sgRNA plasmids, which were transfected into SW480 cells with Lipo3000 transfection reagent. After transfection for 72 h, the GFP-positive cells were subjected to single-cell sorting into 96-well plates using flow cytometry (BD Biosciences, Franklin Lakes, NJ, USA) to ensure monoclonal cell clonality. The knockout cell lines HIF-1α-KO, HIF-2α-KO, and ATX-KO were identified through genomic PCR, DNA sequencing, and western blotting.

### 4.4. Small Interfering RNA Transfection

Small interfering RNAs (siRNAs) targeting HIF-1α, HIF-2α, CBP, and p300 were synthesized by GenePharma Company (Shanghai, China). The sequences of siRNAs are as follows: siHIF-1α, 5′-GCCGCTCAATTTATGAATATT-3′; siHIF-2α, 5′- GCAAATGTACCCAATGATA-3′; siCBP#1, 5′-GCAAACAUCAGUGGGAAUU-3′; siCBP#2, 5′-GCAGCAGCCAGCAUUGAUA-3′; sip300#1, 5′-GCACAAAUGUCUAGUUCUU-3′; sip300#2, 5′-GCCAAUUGCUCACUGCCAU-3′. To knockdown the target genes, siRNAs (20 nM) were transfected into cells with Lipofectamine 3000 according to the manufacturer’s suggestion. The knockdown efficiency of each siRNA was determined using RT-qPCR and/or western blotting.

### 4.5. RNA Isolation and RT-qPCR

Total RNA was isolated from cells using TRIzol reagent (Magen, Guang Zhou, China, #R4801-02) according to the manufacturer’s protocol. Reverse transcription (RT) was performed with 2 μg of total RNA utilizing oligo dT primers (Promega, Madison, WI, #M1705). SYBR green (Yeasen, Shang Hai, China, #11199ES08) quantitative PCR was performed, and the relative expression of each target gene was estimated by normalization to the expression level of β-actin. PCR primers were designed according to published sequences in GenBank. The primer pairs used for qPCR were 5′-AGGCAGTTCCATTCCTGTTC-3′ and 5′-AGGCTGGTGAGATGTTCAAT-3′ for ATX mRNA; 5′-AGAAAATCTGGCACCACACC-3′ and 5′-AGAGGCGTACAGGGATAGCA-3′ for β-actin mRNA; 5′-GTGCTGGCTGAGACCCTAAC-3′ and 5′-GGCTGTCCAAATGGACTTGT-3′ for CBP mRNA; and 5′-CAATGAGATCCAAGGGGAGA-3′ and 5′-ATGCATCTTTCTTCCGCACT-3’ for p300 mRNA.

### 4.6. Chromatin Immunoprecipitation (ChIP) Assays

ChIP assays were performed as described previously [35]. The following sets of primers were used for PCR amplification: ATX promoter site 1, 5′-CACTGGACCTGCTGAGGAAG-3′ (sense) and 5′-AGCAATTTGGCAACCAGCAC-3′ (antisense); ATX promoter site 2, 5′- CCTTGCACAGCCCTGTTTTC-3′ (sense) and 5′- TGGCAACAGGAGGGTTTGTT-3′ (antisense); ATX promoter site 3, 5′-GTCTGTCAACCTCCCAGGTG-3′ (sense) and 5′- TTGCAGCGTGTTCTCTTTGC-3′ (antisense); ATX HRE-1, 5′-GAACGGTTAACAGAAATAGTGGCTG-3′ (sense) and 5′-AAAGAGTTGACCTCAGCCACTA-3′ (antisense); and ATX HRE-2, 5′-CACTGTTGTATGATTGCAAGGGG-3′ (sense) and 5′-AGCCCCTCTACTTGGATTGG-3′ (antisense).

### 4.7. Western Blotting Analysis

The cells were lysed in RIPA buffer (Macgene, Beijing, China, #MP015) supplemented with a protease inhibitor (MedChemExpress, Monmouth Junction, NJ, USA, HY-K0012). After determining the protein concentration using a BCA Protein Assay Kit (Thermo Fisher Scientific, Bremen, Germany, #23227), equivalent protein quantities were subjected to SDS–PAGE and then transferred to PVDF membranes (Millipore, Darmstadt, Germany, #GVWP02500). The membranes were blocked with 5% nonfat milk for 1 h at room temperature and then probed with the indicated primary antibodies, followed by the appropriate HRP-conjugated anti-mouse/rabbit secondary antibodies (Zsgb, Beijing, China, #ZB-2301). Immunoreactive bands were measured with an enhanced chemiluminescence western blotting system (Millipore, Darmstadt, Germany, #WBKLS0500). For the detection of ATX, a secreted protein, cells were cultured in serum-free medium. The medium was concentrated (20-fold) using an Amicon Ultra 30000 (Merck KGaA, Darmstadt, Germany), and then subjected to SDS-PAGE and western blotting.

### 4.8. Luciferase Reporter Gene Assay

The oligonucleotides (~60 bp) derived from each hypoxia response element (HRE) in the ATX promoter were synthesized and inserted into the pGL3-basic vector (Promega, Madison, WI, USA, #E1741) between the M*lu* I and X*hol* I sites to create reporter plasmids for HRE1, HRE2, and HRE3. The core sequences of HRE in the reporter plasmids were mutated from CGT to AAA to construct the corresponding reporter plasmids with mutant HRE1, HRE2, and HRE3. SW480 cells were transfected with the reporter plasmids and then subjected to hypoxic conditions. Luciferase assays were performed with the Dual-Luciferase Reporter Assay System (Promega, Madison, WI, USA, #E1910) according to the manufacturer’s protocol.

### 4.9. Cell Migration Assay

Cell migration was determined using 24-well chambers (Corning, Darmstadt, Germany, #3422) with 8 µm pore polycarbonate membranes coated with collagen I. Parental or ATX-KO SW480 cells (5 × 10^4^) suspended in 200 μL of serum-free IMDM were added to the upper chamber, with 5% FBS IMDM medium added to the lower chamber, and exposed to 21% O_2_ or 1% O_2_ at 37 ℃. The ATX inhibitor PF8380 (10 μM) or 18:1 LPA (2 μM) was added to the medium in the upper and lower chambers as indicated. After 24 h, the nonmigrated cells on the top chambers were removed, and the cells that migrated to the lower side of the upper chamber were fixed with 4% formaldehyde and then stained with a 0.05% hematoxylin solution for 30 min. The cells per microscopic field (SW480 cells, 20×) were imaged and counted in three randomly chosen fields.

### 4.10. Statistical Analysis

Group differences were analyzed using a two-tailed Student’s *t* test between two groups or a two-way ANOVA with Tukey’s *t* test within multiple groups and expressed as the mean ± SEM. Data were collected from at least three independent experiments. The data were then analyzed with the GraphPad Prism 8 software. *p* < 0.05 was considered significant.

## Data Availability

The corresponding author, JJ Zhang, will make all the data behind the study’s conclusions available to the public under reasonable request.

## Supplementary Materials

The following supporting information can be downloaded at: https://www.mdpi.com/article/10.3390/ijms24087031/s1.
