# Supplementary material for: Hypoxia Increases ATX Expression by Histone Crotonylation in a HIF-2α-Dependent Manner
Source: Int J Mol Sci. 2023 Apr 11;24(8):7031. doi: 10.3390/ijms24087031 (PMC10138485; doi:10.3390/ijms24087031)
Supplement: Supplementary file 1 [file ijms-24-07031-s001.zip › ijms-2226779-supplementary.pdf]

Supplementary Figures S1 –S7

Hypoxia increases ATX expression by histone crotonylation in a hypoxia-inducible Factor 2α-dependent manner

Mengxia Qu et al.

*Correspondence: jjzhang@bnu.edu.cn

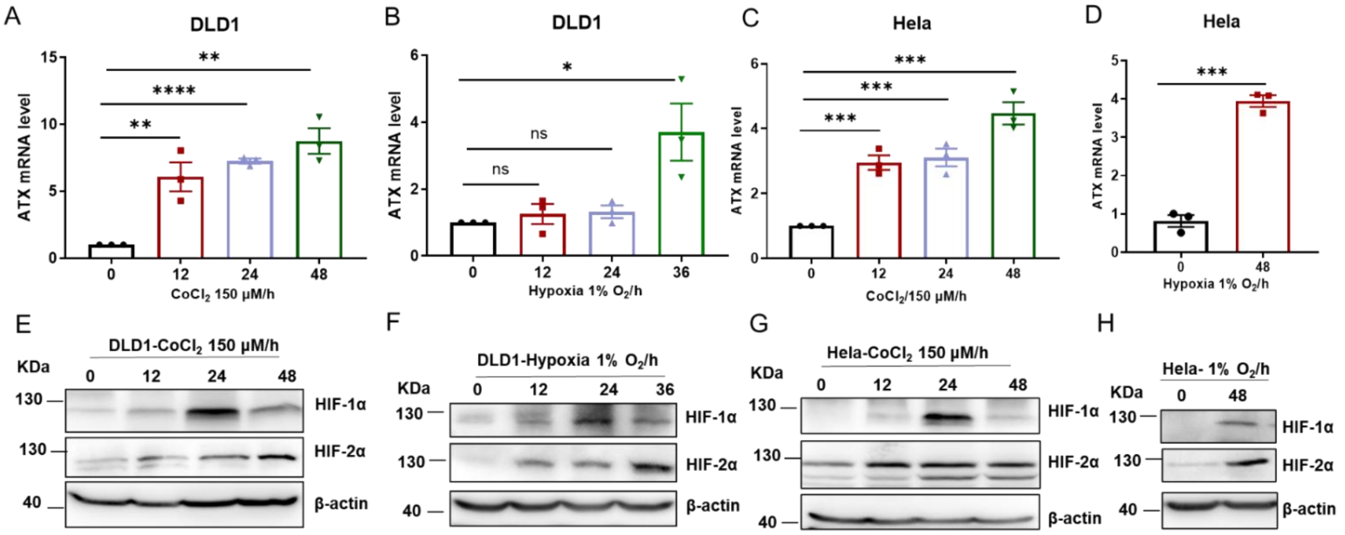

Figure S1. ATX induction by $CoCl_2$ treatment and hypoxia in DLD1 and Hela cells.

(A-D) RT–qPCR analysis of ATX mRNA levels in DLD1 and Hela cells treated with $CoCl_2$ (150 μM) (A,C) or exposed to hypoxia (1% O_2) (B, D) for the indicated times. **(E-H)** Immunoblot assays of HIF-1α and HIF-2α expression levels in DLD1 and Hela cells treated with $CoCl_2$ (150 μM) (E, G) or exposed to hypoxia (1% O2) (F, H) for the indicated times.The data shown are the mean ± SEM of n = 3 independent experiments. *P* values were calculated using two-sided unpaired Student's *t* tests (A-D). ns, not significant; *$p < 0.05$, **$p < 0.01$, ***$p < 0.001$, ****$p < 0.0001$.

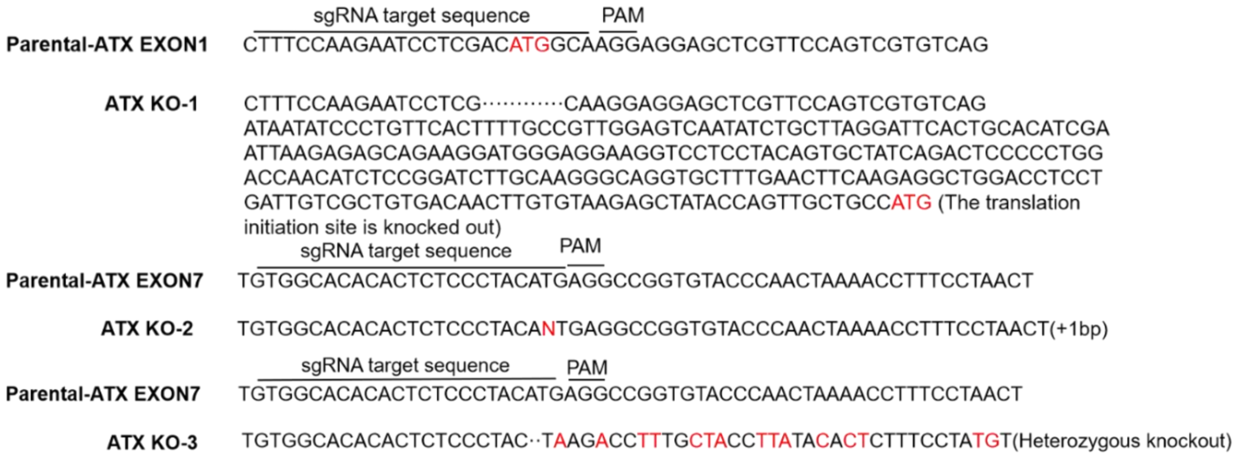

Figure S2. Identification of SW480 cells with ATX knockout (ATX-KO).

ATX-knockout cell lines (ATX KO-1/2/3) were constructed using the CRISPR–Cas9 method with SW480 as parental cells. ATX KO-1/2/3 cells were identified by genomic PCR and DNA sequencing. The nucleotide sequences around gene editing sites are shown.

sgRNA target sequence PAM

Parental-HIF-1α EXON2 CCTCTGTGATGAGGCTTACCATCAGCTATTTGCGTGTGAGGAAACTTCTGGATGCTG

HIF-1α KO CCTCTGTGATGAGGCTTACCATCAGCTATTTGCGTTGTGAGGAAACTTCTGGATGCTG (+1bp)

PAM sgRNA target sequence

Parental-HIF-2α EXON12 GCCCATGTCCTCCATCTTCTTTGATGCCGGAAGCAAAGCATCCCTGCCACCGTGCTGTG

HIF-2α KO GCCCATGTCCTCCA········CTTTGATGCCGGAAGCAAAGCATCCCTGCCACCGTGCTGTG(-4bp)

Figure S3. Identification of SW480 cells with HIF-1α knockout (HIF-1α-KO) and HIF-2α knockout (HIF-2α-KO).
HIF-1α and HIF-2α knockout cell lines were constructed using the CRISPR–Cas9 method with SW480 as parental cells. HIF-1α-KO and HIF-2α-KO cells were identified by genomic PCR and DNA sequencing. The nucleotide sequences around gene editing sites are shown.

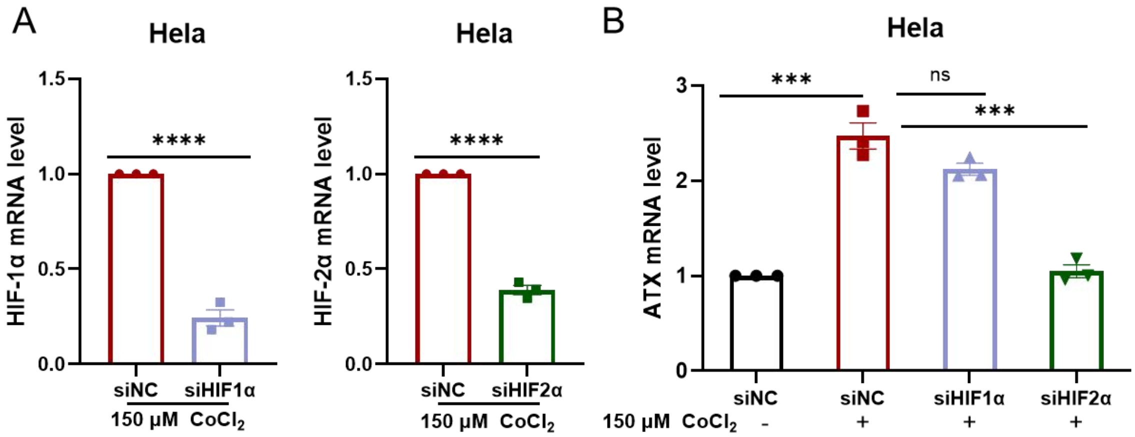

Figure S4. The induction of ATX by $CoCl_2$ treatment is HIF-2α dependent in Hela cells.

(A, B) Hela cells were transfected with HIF-1α siRNA or HIF-2α siRNA, and then treated with $CoCl_2$ (150 μM) for 24 h. HIF-1α, HIF-2α (A) and ATX (B) mRNA levels were detected by RT-qPCR. The data shown are the mean ± SEM of n = 3 independent experiments. P values were calculated using two-sided unpaired Student's *t* tests. ns, not significant, ***$p < 0.001$, ****$p < 0.0001$.

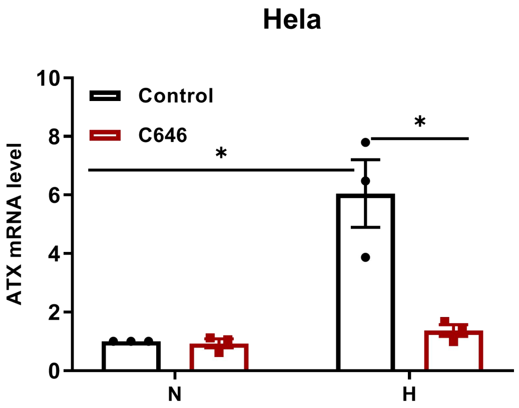

Figure S5. p300 participates in the ATX induction by hypoxia in Hela cells.

Hela cells were cultured under normoxic (N) or hypoxic (1% O2, H) conditions in the absence or presence of the p300 inhibitor C646 (25 μM) for 24 h, and then ATX mRNA levels were detected by RT–qPCR. The data shown are the mean ± SEM of n = 3 independent experiments. P values were calculated using two-sided unpaired Student's *t* tests. *$p < 0.05$.

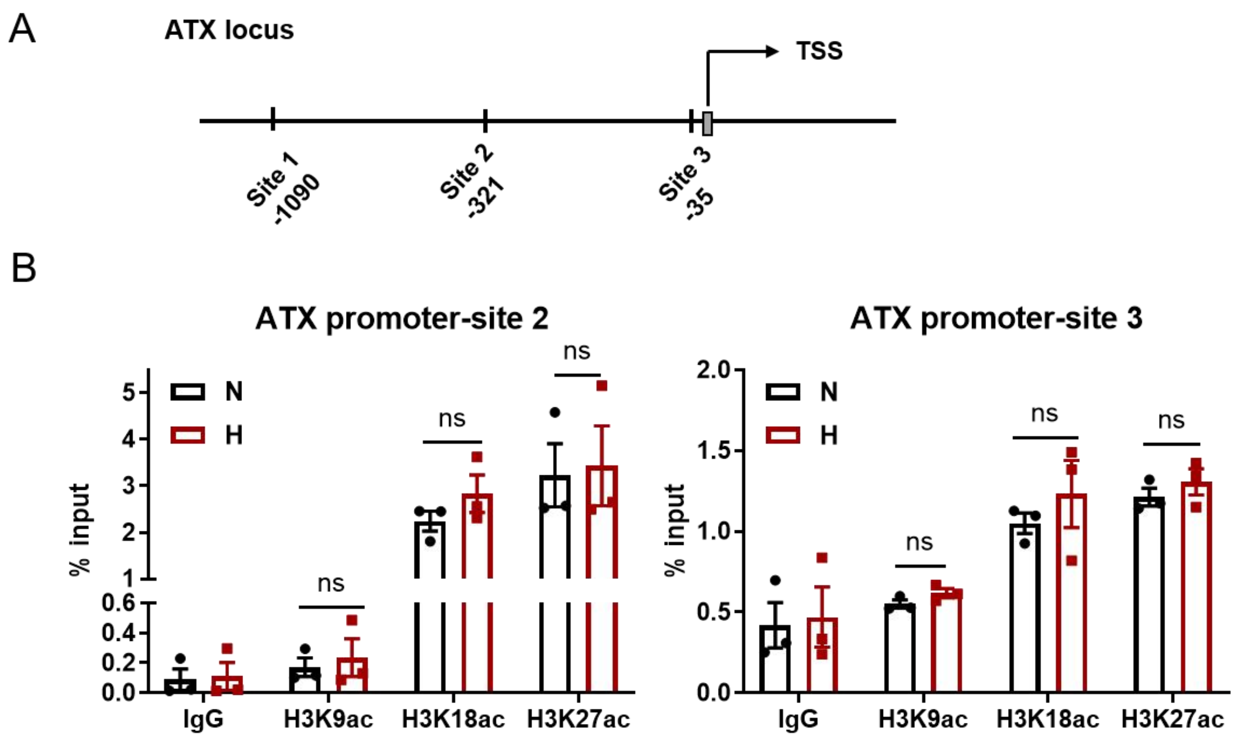

Figure S6. Histone acetylation levels in ATX promoter region in the normoxic and hypoxic SW480 cells.

(A) Schematic diagram of the ChIP–qPCR primer positions. TSS represents the ATX transcription start site. (B) SW480 cells were cultured under normoxic (N) or hypoxic (1% O_2, H) conditions for 24 h and then subjected to ChIP assays with anti-H3K9ac, anti-H3K18ac, or anti-H3K27ac antibodies to detect the enrichment of the indicated histone modifications in the ATX promoter. The immunoprecipitated DNA fragments were subjected to PCR amplification with primers for site 2 and site 3. The data shown are the mean ± SEM of n = 3 independent experiments. *P* values were calculated using two-sided unpaired Student's *t* tests. ns, not significant.

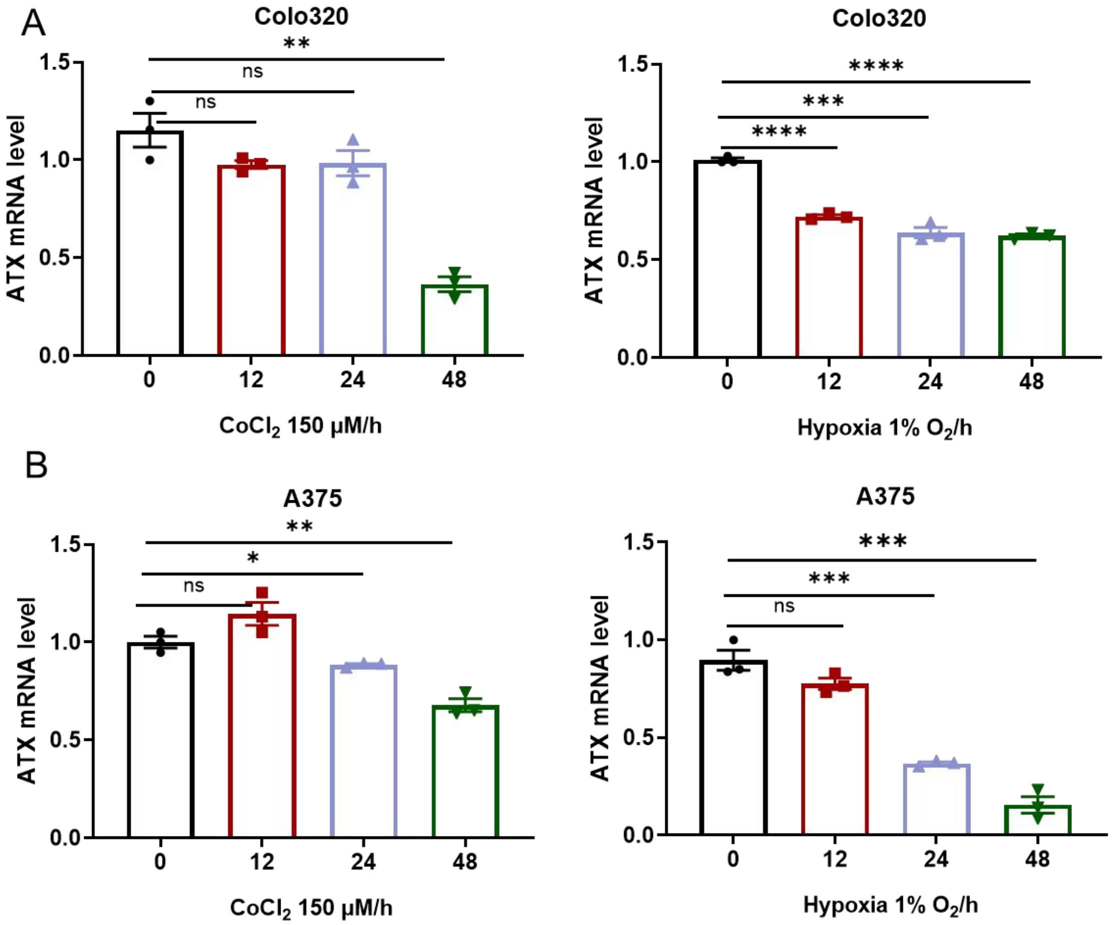

Figure S7. ATX expression is inhibited in Colo320 and A375 cells by $CoCl_2$ treatment and hypoxia.

Colo320 (A) and A375 (B) cells were treated with $CoCl_2$ (150 µM) or exposed to hypoxia (1% O_2) for 0, 12, 24 or 48 h as indicated, and then ATX mRNA levels were detected by RT–qPCR. The data shown are the mean ± SEM of n = 3 independent experiments. P values were calculated using two-sided unpaired Student's *t* tests (A, B). ns, not significant, *$p < 0.05$, **$p < 0.01$, ***$p < 0.001$, ****$p < 0.0001$.
